# Analysis of Proanthocyanidins in Plant Materials Using Hydrophilic Interaction HPLC-QTOF-MS

**DOI:** 10.3390/molecules27092684

**Published:** 2022-04-21

**Authors:** Ziqi Qian, Yi Wang, Shuyu Shen, Wenyang Tao, Yu Zhang, Xingqian Ye, Shiguo Chen, Haibo Pan

**Affiliations:** 1 National-Local Joint Engineering Laboratory of Intelligent Food Technology and Equipment, Zhejiang Key Laboratory for Agro-Food Processing, Zhejiang Engineering Laboratory of Food Technology and Equipment, College of Biosystems Engineering and Food Science, Zhejiang University, Hangzhou 310058, China; 21913041@zju.edu.cn (Z.Q.); jssqwangyi@163.com (Y.W.); wytao@zju.edu.cn (W.T.); psu@zju.edu.cn (X.Y.); chenshiguo210@163.com (S.C.); 2 Fuli Institute of Food Science, Zhejiang University, Hangzhou 310058, China; 3 Natural Medicine Institute of Zhejiang YangShengTang Co., Ltd., No. 181, Geyazhuang, Xihu District, Hangzhou 310058, China; vera_shensy@163.com; 4 School of Medical Instruments and Food Engineering, University of Shanghai for Science and Technology, Shanghai 200093, China; zyu20@usst.edu.cn; 5 Ningbo Research Institute, Zhejiang University, Ningbo 315100, China; 6 Zhongyuan Institute, Zhejiang University, Zhengzhou 450000, China

**Keywords:** Chinese bayberry leaves, sorghum testa, grape seeds, proanthocyanidins, HILIC-QTOF-MS

## Abstract

Proanthocyanidins (PACs) have been proven to possess a wide range of biological activities, but complex structures limit their study of structure–function relationships. Therefore, an efficient and general method using hydrophilic interaction high-performance liquid chromatography coupled with high-resolution quadrupole time-of-flight tandem mass spectrometry (HILIC-QTOF-MS) was established to analyze PACs from different plant materials. This method was successfully applied to characterize PACs from Chinese bayberry (*Myrica rubra* Sieb. et Zucc.) leaves (BLPs), sorghum testa (STPs) and grape seeds (GSPs). BLPs with the degree of polymerization (DP) from 1 to 8 were separated. BLPs are mainly B-type prodelphinidins and A-type BLPs were first found in this study. STPs and GSPs belonging to procyanidins showed DP from 3 to 11 and 2 to 12, respectively. A-type linkages were found for every DP of STPs and GSPs, which were first found. These results showed that HILIC-QTOF-MS can be successfully applied for analyzing PACs from different plant materials, which is necessary for the prediction of their potential health benefits.

## 1. Introduction

Proanthocyanidins (PACs), also known as condensed tannins, are one of the most abundant dietary polyphenols, second to lignin [1]. They comprise oligomeric or polymeric flavan-3-ol monomeric subunits, resulting in their characteristic of high molecular weight [2]. Monomeric subunits linked through C_4_–C_6_ or C_4_–C_8_ bonds generate B-type PACs, and an additional linkage of C_2_–O_7_ forms A-type ones. Based on flavan-3-ol subunits, PACs are further subdivided into procyanidins, prodelphinidins and propelargonidins, with (epi)catechin (EC), (epi)gallocatechin (EGC) and (epi)afzelechin as subunits [3]. In addition, gallic acid esters of the flavan-3-ols are also found to be monomeric subunits of PACs in nature. The number of monomeric subunits dictates the degree of polymerization (DP), which varies greatly depending on the plant sources.

Numerous studies have revealed the vital role of PAC structure in their beneficial effects on health [4,5,6,7,8]. A-type PACs displayed interesting antibacterial and antiviral properties by inhibiting bacterial adhesion and virus replication [4]. As to DP, only oligomeric procyanidins including dimers, trimers and tetramers were absorbable while polymers exhibited extremely low bioavailability with no detectable PACs present in blood circulation [5,6]. In the case of prodelphinidins, only dimer was absorbable as demonstrated with Caco-2 cell permeability assays [7]. PAC with a mean DP of 9.1 regulated inflammatory cytokine responses in murine macrophages, whereas others were significantly less active [8]. In light of the structure–function relationships of PACs, it is necessary to analyze their structure to predict potential health benefits.

Reverse-phase high-performance liquid chromatography (RP-HPLC) is most commonly used to analyze PACs, but polymers with DP over four are difficult to be identified due to their high polarity and many isomers, which lead to the increase in baseline [9]. Normal-phase HPLC (NP-HPLC) was able to separate polymeric PACs based on the DP [10,11]. However, it uses solvents with low polarity such as hexane and hexamethylene as mobile phases, which have low intermolecular dispersion, resulting in difficult ionization. Recently, hydrophilic interaction chromatography (HILIC), retaining analytes by partitioning between the water layer on the hydrophilic stationary phase and the polar eluent, has attracted increasing attention [12]. HILIC coupled with a fluorescence detector has been established to separate PACs with EC as exclusive subunits based on their DP [13]. However, the HILIC method is not feasible to analyze PACs containing monomeric subunits except EC. In addition, the fluorescence detector limits the popularization of the HILIC method.

Hence, the objective of this study is to establish a widely used method to analyze PACs from different plant materials using HILIC-QTOF-MS, which is appropriate for batch analysis. This method was successfully applied to separate and characterize PACs from Chinese bayberry (*Myrica rubra* Sieb. et Zucc.) leaves (BLPs), sorghum testa (STPs) and grape seeds (GSPs).

## 2. Results and Discussion

**Figure 1:**
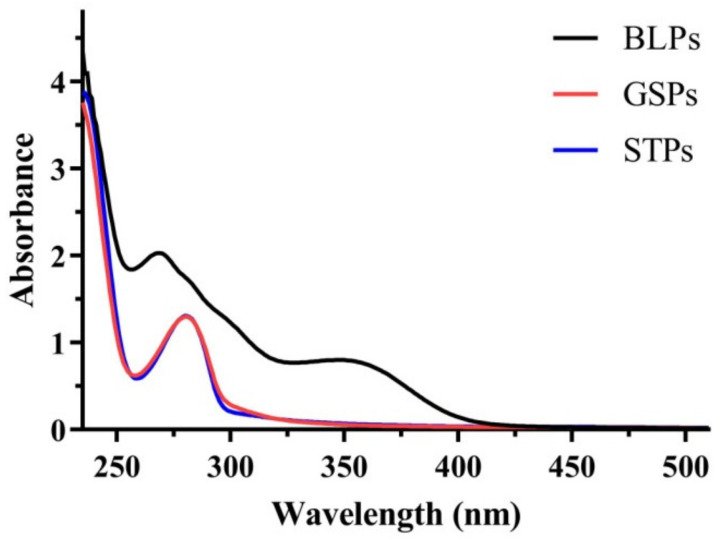
UV–Vis spectra of Chinese bayberry (Myrica rubra Sieb. et Zucc.) leaves (BLPs), sorghum testa (STPs) and grape seeds (GSPs).

To reveal the differences in BLPs, GSPs and STPs in molecular structure, UV/Visible spectrum analysis was first carried out. As shown in Figure 1, the UV/Visible spectra of BLPs, GSPs and STPs exhibited a maximum absorption wavelength of 268, 280 and 280 nm, which were in agreement with the main absorption maxima of natural phenolic compounds [14,15]. In addition, a shoulder peak at 328 nm appeared in the UV/Visible spectrum of BLPs, while the peak did not exist in GSPs and STPs. The results of UV/Visible spectrum analysis indicated that GSPs and STPs had the same subunits, which were different from BLPs.

To verify the speculation, HILIC-QTOF-MS/MS was performed to analyze the detailed structures of BLPs, GSPs and STPs. Identification of the PACs depends on analyzing the MS data of the peaks separated by HILIC. The molecular weight of PACs is determined by flavan-3-ol subunits, linkage type and DP. The molecular weight of the flavan-3-ols is definite. Each A-type linkage leads to the loss of four hydrogen atoms, while B-type linkage results in the loss of two hydrogen atoms. In addition, the type of flavan-3-ol subunits is limited, as less than four types of flavan-3-ols usually appear in PACs from individual plant material. According to the description above, identification of the PACs with MS data becomes possible. MS/MS data were used to verify the results of MS data. The MS main ions generated MS/MS product ions with several fragmentation routes, including quinone methide (QM) cleavage, retro-Diels–Alder (RDA) cleavage, heterocyclic ring fission (HRF), gallate loss (GL) and benzofuran formation (BFF) [16,17,18]. In addition, GL combined with GL, HRF, BFF or RDA cleavage generated a series of product ions [19].

**Figure 2:**
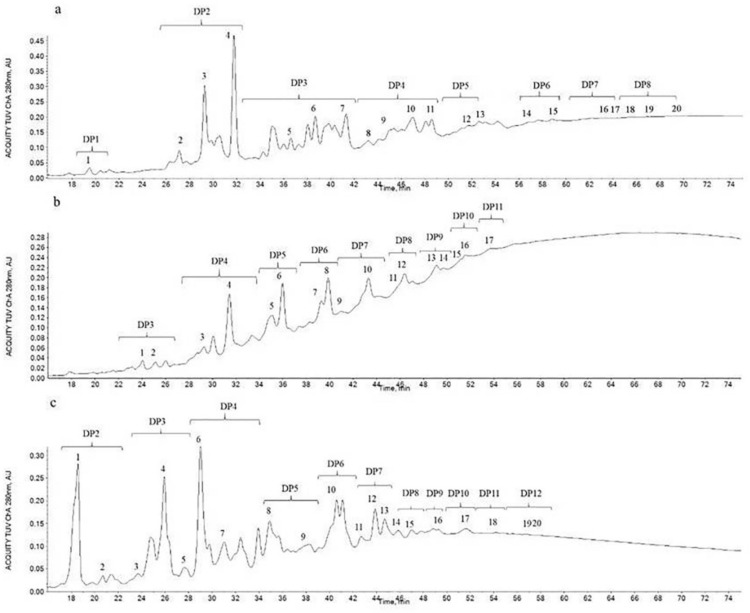
Chromatographic separation of BLPs (**a**), STPs (**b**) and GSPs (**c**), according to the DP.

The HILIC chromatogram was given in Figure 2. HILIC indeed was able to separate PACs based on their DP, but resolution of the peaks varied with the distribution of their DP and isomers. The baseline had an upward drift along with the retention time, which resulted from the low resolution of PACs with high DPs (Rue et al., 2018) [12]. The detailed MS information of HILIC peaks is shown in Table 1, Table 2 and Table 3. BLPs showed the DP from 2 to 8, STPs showed the DP from 3 to 11 and GSPs showed the DP from 2 to 12. Various isomers of each DP were identified.

As shown in Table 1, BLPs are mainly B-type prodelphinidins with (epi)gallocatechin gallate ((E)GCG) as dominant subunits and (E)GC as minor subunits, which was consistent with the results of our previous article [20]. However, only dimers, trimers and tetramers were identified in the reported article. In the present study, BLPs with higher DP from 5 to 8 corresponding to the peaks from 12 to 20 were first identified. Peaks 12 and 13 were tentatively identified as a B-type pentamer. Peak 12 consisting of two (E)GC units and three (E)GCG units produced fragment ions at *m*/*z* 760.1393, 1217.2235 and 1522.2688 by QM fragmentation, while ion at *m*/*z* 423.0715 was obtained by RDA fragmentation. Peak 13 consisting of one (E)GC unit, and four (E)GCG units produced a similar fragmentation pattern in the MS/MS spectrum. Peaks 14 and 15 were all presumed as B-type hexamer, the former consisting of two (E)GC units, and four (E)GCG units produced a double charged pseudomolecular ion [M-2H]^2−^ at *m*/*z* 1216.1920, the latter consisting of six (E)GCG units produced a double charged pseudomolecular ion [M-2H]^2−^ at *m*/*z* 1368.1997. Peaks 16 and 17 were assigned as B-type heptamer with one (E)GC unit and six (E)GCG units, and seven (E)GCG units, respectively. Peaks 18, 19 and 20 were all presumed as octamers with two (E)GC units and six (E)GCG units, one (E)GC unit and seven (E)GCG units, and eight (E)GCG units, respectively. In addition, A-type BLPs including a dimer (peak 2) and a tetramer (peak 8) were found in the present study, which was not reported before. The A-type dimer consisted of one (E)GC unit and one (E)GCG unit. The A-type tetramer comprised two (E)GC units and two (E)GCG units, which seemed to have resulted from the loss of two hydrogens of peak 9.

The composition of STPs and GSPs, which was determined by their retention time, pseudomolecular ion [M-H]^−^ and MS/MS information, was given in Table 2 and Table 3. STPs and GSPs were procyanidins with (E)C as only subunits. STPs with the DP from 3 to 11 and GSPs with the DP from 2 to 12 were isolated. STPs were found to contain A-type linkages in this study. Interestingly, GSPs contained many A-type linkages, which is not consistent with previous studies [20]. The reason for it is not clear, and it may be due to the variety of samples.

In the HILIC chromatogram of STPs, Peaks 1 and 2, identified as trimers, produced fragment ions at *m*/*z* 407.0767 and 411.0726 by RDA fragmentation and successive loss of water molecules, whereas ion at *m*/*z* 289.0709 was obtained by QM fragmentation. In addition, the loss of 2 Da in peak 1 is due to the additional C-O-C linkage. Peak 4 was assigned as a B-type tetramer. It produced fragment ions at *m*/*z* 865.2100, 575.1238 and 287.0553 by QM fragmentation and successive loss of water molecules, whereas ion at *m*/*z* 739.1748 was obtained by HRF, and ion at *m*/*z* 407.0780 was obtained by RDA fragmentation and successive loss of water molecules. Peak 3, with a similar MS/MS pattern, was tentatively identified as an A-type tetramer. Peaks 5 and 6 were tentatively identified as a pentamer, and peak 5 contains one A-type linkage. The produced fragment ion at *m*/*z* 407.0780 was obtained by RDA fragmentation and successive loss of water molecules, whereas ion at *m*/*z* 289.0703 was obtained by QM fragmentation. Peaks 7 and 8 were tentatively identified as a hexamer, and peak 7 contains one A-type linkage. Peak 10, producing fragment ions at *m*/*z* 863.1896, 575.1197 and 287.0539 by QM fragmentation, was identified as a B-type heptamer. Peak 9, with a similar MS/MS pattern, was tentatively identified as an A-type heptamer. Peak 11 was assigned as a B-type octamer. It produced fragment ions at *m*/*z* 1728.3798, 1440.3280, 1152.2677 and 865.2083 by QM fragmentation, whereas ion at *m*/*z* 693.1234 was obtained by RDA fragmentation and successive loss of water molecules. Peak 12, with a similar MS/MS pattern, was tentatively identified as an A-type octamer. Peaks 13 to 17 were detected as polymers with the DP from 9 to 11. Among them, Peaks 14 and 15 contain one A-type linkage. In addition, to our knowledge, this is the first report identifying STPs with a DP of 11, even though the STPs with high DP were well-known.

The composition of GSPs is similar to that of STPs, but GSPs have more A-type linkages. In the HILIC chromatogram of GSPs, Peak 3 was tentatively identified as a trimer containing two A-type linkages. It produced fragment ions at *m*/*z* 285.0398 and 571.0902 by QM fragmentation. Peaks 6, 8, 10, 13, 15 and 16, with a similar MS/MS pattern, were tentatively identified as tetramer to nonamer with two A-type linkages. Peak 12 was tentatively assigned to a heptamer with three A-type linkages. It produced fragment ions at *m*/*z* 285.0426 and 575.1219 by QM fragmentation, whereas ion at *m*/*z* 411.0749 was obtained by HRF. Peaks 14, 17, 18 and 20, with a similar MS/MS pattern, were tentatively identified as polymers with a DP of 8, 10, 11 and 12 containing three A-type linkages. Peak 19 was tentatively assigned to a dodecamer with four A-type linkages. It produced double-charged pseudomolecular ions [M-2H]^2−^ at *m*/*z* 1724.3341.

The obtained structure information of PACs could help to predict their functional characteristics, such as bioavailability and physiological effects. As to BLPs, the small proportion of dimers in BLPs indicated their low bioavailability. In the case of procyanidins, GSPs have more oligomers than STPs, suggesting higher bioavailability of GSPs than STPs. It is worth noticing that the large number of A-type linkages in STPs endows their unique physiological effects.

BLPs, GSPs and STPs, polymers with high polarity and have many isomers, are difficult to be identified by traditional RP-HPLC, due to the increase in baseline when analyzing compounds with a DP more than four. The traditional NP-HPLC is rarely used to separate proanthocyanidins, because of their low solubility in organic solvents, strong silica gel adsorption and difficult ionization. Emerging HILIC is popular for separation of proanthocyanidins, but it is not feasible to analyze proanthocyanidins containing monomeric subunits, except for epicatechin, and fluorescence detector limits its popularization. Overall, the HILIC-QTOF-MS method established in the present study is a widely used method to analyze PACs from different plant materials, which is helpful for the in-depth study of their structure–function relationships.

## 3. Materials and Methods

### 3.1. Materials and Reagents

Chinese bayberry leaves of ‘Biqi’ cultivar were hand-harvested randomly in June, 2020 in Cixi (Zhejiang, China). Sorghums of ‘Hongzhenzhu’ cultivar were provided by Shandong Academy of Agricultural Science (Jinan, China). GSPs (95%) were purchased from Shanghai Yuanye Biotechnology Co., Ltd. (Shanghai, China). Acetonitrile and methanol of HPLC grade were purchased from Merck KgaA (Darmstadt, Germany). Acetic acid of HPLC grade was purchased from Aladdin Biochemical Technology Co., Ltd. (Shanghai, China). Other chemical reagents of analytical grade were purchased from Sinopharm Chemical Reagent Co., Ltd. (Shanghai, China).

### 3.2. Extraction and Purification of PACs

Extraction and purification of BLPs and STPs were carried out according to our previous studies [20]. In brief, Chinese bayberry leaves were dried at 40 °C for 12 h and then ground well into a powder by milling. Powder of sorghum testa was prepared by a rice polisher (SATAKE Manufacturing Co., Ltd., Suzhou, China) and then passed through a 500 μm sieve. Obtained powders (50 g) were extracted with 70% aqueous acetone containing 0.1% (*w*/*v*) ascorbic acid (500 mL) at room temperature for 12 h. The extract solution was recovered and washed with hexane and dichloromethane. Then, organic solvent was evaporated by rotary evaporation and residual aqueous phase was freeze-dried to crude extract of PACs, which was then purified by the HPD-500 resin to remove proteins and polysaccharides with water as an elution solvent. PACs were then eluted with 80% ethanol and dried by rotary-evaporated under vacuum to remove organic solvent and lyophilized to a brown powder. PACs were further purified with a Sephadex LH-20 column (300 mm × 30 mm i.d.). The column was equilibrated with a methanol/water solution (1:1, *v*/*v*) containing 0.1% *v*/*v* trifluoroacetic acid. The brown powder (2.0 g) was dissolved in the mobile phase and loaded onto the column. The column was first eluted with 3 column volumes of the mobile phase to remove free flavan-3-ols. PACs were then eluted with 3 column volumes of an acetone/water solution (2:1 *v*/*v*) containing 0.1% *v*/*v* trifluoroacetic acid and the eluent of PACs was collected. The eluent was concentrated under reduced pressure at 40 °C to remove methanol and acetone and then lyophilized to dry powder.

### 3.3. UV–Vis Spectroscopic Measurement

UV–Vis spectra of BLPs, GSPs and STPs were recorded at room temperature over the wavelength range of 200 to 800 nm using a UV–vis spectrometer (UV-2600, Shimadzu Co., Kyoto, Japan). The methanol was used as a background.

### 3.4. HPLC-QTOF-MS Analysis

Chromatographic separation of PACs was performed with a X5H HILIC column (250 mm × 4.6 mm, 5.0 μm, Acchrom Technologies, Taizhou, China). PACs were dissolved in methanol and then filtered through 0.45 μm membrane filter for HPLC analysis (Waters, Milford, CT, USA). The binary mobile phase consisted of (A) acetic acid/acetonitrile (0.1/99.9, *v*/*v*) and (B) acetic acid/water/methanol (0.1/3/96.9, *v*/*v*/*v*). The flow rate was 0.5 mL/min and the gradient conditions were as follows: 0–3 min, 7% linear; 3–15 min, 7–23% linear; 15–70 min, 23–65% linear; 70–85 min, 65–100% linear; 85–87 min, 100–7% linear; 87–102 min, 7% linear. Eluting peaks were monitored at 280 nm. The column temperature was kept at 28 °C.

MS ionization was operated in negative mode on Triple TOF 5600plus System (AB SCIEX, Framingham, MA, USA) using the following conditions: scan range, *m*/*z* 100–2000; source voltage, −4.5 kV; and source temperature, 550 °C. The pressure of ion source gas 1 (Air), ion source gas 2 (Air) and curtain gas (N_2_) were set at 50 psi, 50 psi and 30 psi, respectively. Injection volume was set at 10 μL. Flow rate was set at 0.2 mL/min. Maximum allowed error was set at ±5 ppm. Declustering potential was set at 100 V. Collision energy was set at 10 V. For MS/MS acquisition mode, the IDA-based auto-MS/MS was performed on the 8 most intense metabolite ions, the parameters were almost the same except that the collision energy was set at −40 ± 20 V, ion release delay at 67 and ion release width at 25 in a cycle of full scan (1 s). The scan range of *m*/*z* of precursor ion and product ion was set at 100–2000 Da and 50–1500 Da, respectively.

## 4. Conclusions

The structure of PACs from different sources is significantly different, which brings difficulties to their structure–activity study. A novel and a general method were exhibited in this study to analyze PACs from different sources using HILIC-QTOF-MS, which is more efficient than other conventional methods, especially for polymers. It was found that BLPs are mainly B-type prodelphinidins with (E)GCG as dominant subunits and (E)GC as minor subunits. BLPs with DP from 1 to 8 were separated effectively and few A-type linkages were found. STPs are procyanidins with (E)C as exclusive subunits. STPs with DP from 3 to 11 could be separated and almost each DP contains one A-type linkage. GSPs are also procyanidins with DP from 2 to 12 and every DP contains A-type linkages with the most up to four. Hence, the HILIC-QTOF-MS method established in this study can be applied to analyze large numbers of PACs from different sources, which is necessary for prediction of their potential health benefits.

## Figures and Tables

**Table 1:** Main prodelphinidins identified from Chinese bayberry leaves using HILIC-QTOF-MS.

Peak	DP	Retention Time (min)	Molecular Formula	N_A_	[M-H]^−^	[M-2H]^2−^	[MS/MS]	Error (ppm)	Identification
1	1	19.561	C_22_H_18_O_11_	-	457.0776	-	125.0249HRF, 169.0143(gallic acid)	0.7	(E)GCG
2	2	27.206	C_37_H_28_O_18_	1	759.1228	-	177.0204HRF, 301.0366QM, 455.0648RDA	0.4	(E)GC + (E)GCG
3	2	29.275	C_37_H_30_O_18_	-	761.1362	-	125.0249HRF, 177.0195HRF, 305.0671QM, 423.0730RDA	−0.3	(E)GC + (E)GCG
4	2	31.659	C_44_H_34_O_22_	-	913.1470	-	177.0194HRF, 285.0404QM, 423.0726RDA, 591.1177(GL, galloyl loss)	0.9	2(E)GCG
5	3	36.757	C_52_H_42_O_25_	-	1065.1929	-	177.0198HRF; 243.0298; 423.0740RDA	−1	2(E)GC + (E)GCG
6	3	39.413	C_59_H_46_O_29_	-	1217.2025	-	423.0742RDA, 709.1262(RDA, galloyl loss), 1047.1941(galloyl loss)	−2.3	(E)GC + 2(E)GCG
7	3	41.382	C_66_H_50_O_33_	-	1369.2128	684.1023	125.0266HRF, 169.0156(gallic acid), 285.0411QM, 423.073RDA	−1.4	3(E)GCG
8	4	44.517	C_74_H_56_O_36_	1	1519.2421	759.1204	-	−2.2	2(E)GC + 2(E)GCG
9	4	44.998	C_74_H_58_O_36_	-	1521.2564	760.1275	125.0266HRF, 169.0157(gallic acid), 177.0211HRF, 243.0307	−3.5	2(E)GC + 2(E)GCG
10	4	47.107	C_81_H_62_O_40_	-	1673.2664	836.1304	125.0272HRF, 169.0162(gallic acid), 177.0214HRF, 319.0479	−2.9	(E)GC + 3(E)GCG
11	4	48.637	C_88_H_66_O_44_	-	1825.2773	912.1391	169.0159(gallic acid), 177.0229HRF, 285.0426QM, 319.0496	0.5	4(E)GCG
12	5	51.776	C_96_H_74_O_47_	-	1977.3243	988.1548	169.0156(gallic acid), 177.0207HRF, 243.0312; 319.0461; 423.0715RDA, 760.1393QM, 1217.2235QM, 1522.2688QM	−1.6	2(E)GC + 3(E)GCG
13	5	52.447	C_103_H_78_O_51_	-	-	1064.1583	169.0160(gallic acid), 303.0535, 423.0767RDA, 762.1430QM, 1066.2029(two GL), 1370.2245QM	−0.8	(E)GC + 4(E)GCG
14	6	57.218	C_118_H_90_O_58_	-	-	1216.1920	-	−2.1	2(E)GC + 4(E)GCG
15	6	59.001	C_132_H_98_O_66_	-	-	1368.1997	-	−0.6	6(E)GCG
16	7	63.259	C_147_H_110_O_73_	-	-	1520.2345	-	0.5	(E)GC + 6(E)GCG
17	7	64.176	C_154_H_114_O_77_	-	-	1596.2549	-	0.4	7(E)GCG
18	8	66.286	C_162_H_120_O_80_	-	-	1671.2474	-	−1.2	2(E)GC + 6(E)GCG
19	8	67.333	C_169_H_125_O_84_	-	-	1748.7661	-	−3.1	(E)GC + 7(E)GCG
20	8	69.309	C_176_H_130_O_88_	-	-	1824.2760	-	2.4	8(E)GCG

N_A_, number of A-type linkage; (epi)gallocatechin ((E)GC); (epi)gallocatechin gallate ((E)GCG).

**Table 2:** Main procyanidins identified from sorghum testae using HILIC-QTOF-MS.

Peak	DP	Retention Time (min)	Molecular Formula	N_A_	[M-H]^−^	[M-2H]^2−^	[MS/MS]	Error (ppm)	Identification
1	3	23.9703	C_45_H_36_O_18_	1	863.1870	-	289.0709QM, 411.0726(RDA, H_2_O loss)	4.3	3(E)C
2	3	25.0561	C_45_H_38_O_18_	-	865.2014	-	289.0698QM, 407.0767(RDA, H_2_O loss)	3.3	3(E)C
3	4	29.1185	C_60_H_48_O_24_	1	1151.2478	-	287.0547QM, 407.0774(RDA, H_2_O loss), 575.1241QM, 861.1795QM	2.4	4(E)C
4	4	31.4384	C_60_H_50_O_24_	-	1153.2632	-	287.0553QM, 407.0780(RDA, H_2_O loss), 575.1238QM, 739.1748HRF, 865.2100QM	−0.1	4(E)C
5	5	34.8734	C_75_H_60_O_30_	1	1439.3088	719.1500	289.0703QM, 407.0769(RDA, H_2_O loss), 529.0789RDA	−0.8	5(E)C
6	5	35.9701	C_75_H_62_O_30_	-	1441.3215	720.1579	289.0693QM, 407.0757(RDA, H_2_O loss)	−1.4	5(E)C
7	6	39.2811	C_90_H_72_O_36_	1	1728.3641	863.1802	289.0700QM, 411.0707(RDA, H_2_O loss)	−1.3	6(E)C
8	6	39.8536	C_90_H_74_O_36_	-	1729.3784	864.1872	289.0696QM, 407.0755(RDA, H_2_O loss)	−3.8	6(E)C
9	7	41.0480	C_105_H_84_O_42_	1	-	1007.2108	285.0382QM, 449.0887HRF, 575.1190QM	−2.1	7(E)C
10	7	43.2758	C_105_H_86_O_42_	-	-	1008.2181	287.0539QM, 407.0757(RDA, H_2_O loss), 575.1197QM, 863.1896QM	−3.7	7(E)C
11	8	46.2532	C_120_H_98_O_48_	-	-	1152.2455	287.0540QM, 407.0749(RDA, H_2_O loss), 449.0863HRF, 575.1198QM, 693.1234(RDA, H_2_O loss), 865.2083QM, 1152.2677QM, 1440.3280QM, 1728.3798QM	−4.7	8(E)C
12	8	47.1237	C_120_H_96_O_48_	1	-	1151.2415	287.0534QM, 407.0742(RDA, H_2_O loss), 575.1182QM, 693.1235(RDA, H_2_O loss), 861.1725QM, 1439.3156QM, 1726.3827QM	−5.3	8(E)C
13	9	48.9865	C_135_H_110_O_54_	-	-	1296.2752	-	−5.8	9(E)C
14	9	49.7734	C_135_H_108_O_54_	1	-	1295.2678	-	−6.3	9(E)C
15	10	51.1335	C_150_H_120_O_60_	1	-	1439.2981	-	−5.4	10(E)C
16	10	51.5021	C_150_H_122_O_60_	-	-	1440.3040	-	−7.7	10(E)C
17	11	53.4928	C_165_H_134_O_66_	-	-	1584.3329	-	−5.4	11(E)C

N_A_, number of A-type linkage; (epi)catechin ((E)C).

**Table 3:** Main procyanidins identified from grape seeds using HILIC-QTOF-MS.

Peak	DP	Retention Time (min)	Molecular Formula	N_A_	[M-H]^−^	[M-2H]^2−^	[MS/MS]	Error (ppm)	Identification
1	2	18.4672	C_30_H_24_O_12_	1	575.1180	-	285.0392QM, 407.0702(RDA, H_2_O loss)	−2	2(E)C
2	2	20.7521	C_30_H_26_O_12_	-	577.1339	-	289.0711 QM, 407.0792(RDA, H_2_O loss)	−1.9	2(E)C
3	3	23.5959	C_45_H_34_O_18_	2	861.1660	-	285.0398QM, 571.0902QM	−1.5	3(E)C
4	3	25.9204	C_45_H_36_O_18_	1	863.1807	-	285.0402QM, 407.0742(RDA, H_2_O loss), 449.0807HRF	−1.9	3(E)C
5	3	27.7716	C_45_H_38_O_18_	-	865.1951	-	289.0721QM, 407.0702(RDA, H_2_O loss)	−3.2	3(E)C
6	4	29.0211	C_60_H_46_O_24_	2	1149.2240	-	285.0409QM, 411.0733HRF, 575.1215QM, 979.1813(RDA, H_2_O loss)	−4.5	4(E)C
7	4	31.1407	C_60_H_48_O_24_	1	1151.2387	-	287.0576QM, 449.0890HRF, 575.1214QM, 693.1288(RDA, H_2_O loss), 863.1882QM, 981.1936(RDA, H_2_O loss)	−6.1	4(E)C
8	5	34.8175	C_75_H_58_O_30_	2	1437.2789	718.1421	285.0425QM, 411.0735HRF, 575.1183QM	−1.6	5(E)C
9	5	38.3058	C_75_H_60_O_30_	1	1439.2956	719.1503	285.0422QM, 407.0812(RDA, H_2_O loss)	−1.3	5(E)C
10	6	40.6233	C_90_H_70_O_36_	2	1725.3401	861.1636	285.0438QM, 411.0754HRF, 575.1231QM	−5.9	6(E)C
11	6	42.6287	C_90_H_72_O_36_	1	1727.3537	863.1782		−5.8	6(E)C
12	7	43.9009	C_105_H_80_O_42_	3	-	1005.6966	285.0426QM, 411.0749HRF, 575.1219QM	−3.3	7(E)C
13	7	44.7250	C_105_H_82_O_42_	2	-	1006.2002	285.0436QM, 411.0745HRF, 575.1225QM	−4.5	7(E)C
14	8	45.8513	C_120_H_92_O_48_	3	-	1149.2220	287.0584QM, 411.0737HRF, 575.1229QM, 863.1879QM, 1151.2521QM	−6.2	8(E)C
15	8	46.9126	C_120_H_94_O_48_	2	-	1150.2270	287.0580QM, 411.0745HRF, 575.1212QM, 863.1855QM, 1151.2475QM	−7.9	8(E)C
16	9	48.8524	C_135_H_106_O_54_	2	-	1294.2588	-	−8.8	9(E)C
17	10	51.6613	C_150_H_116_O_60_	3	-	1437.2806	-	−9.3	10(E)C
18	11	54.2179	C_165_H_128_O_66_	3	-	1581.3085	-	−9.2	11(E)C
19	12	56.7175	C_180_H_138_O_72_	4	-	1724.3341	-	−5.8	12(E)C
20	12	57.7746	C_180_H_140_O_72_	3	-	1725.3350	-	−9.1	12(E)C

N_A_, number of A-type linkage; (epi)catechin ((E)C).

## Data Availability

Not applicable.

## Sample Availability

Samples of the compounds are available from the authors.
